# CRISPA: A Non-viral, Transient Cas9 Delivery System Based on Reengineered Anthrax Toxin

**DOI:** 10.3389/fphar.2021.770283

**Published:** 2021-10-18

**Authors:** Maximilian Hirschenberger, Nicole Stadler, Maximilian Fellermann, Konstantin M. J. Sparrer, Frank Kirchhoff, Holger Barth, Panagiotis Papatheodorou

**Affiliations:** ^1^ Institute of Pharmacology and Toxicology, Ulm University Medical Center, Ulm, Germany; ^2^ Institute of Molecular Virology, Ulm University Medical Center, Ulm, Germany

**Keywords:** CRISPR/Cas9, genome editing, cell targeting, bacterial toxin, pore-forming toxin, translocation channel, protein translocation

## Abstract

Translating the CRISPR/Cas9 genome editing technology into clinics is still hampered by rather unspecific, unsafe and/or inconvenient approaches for the delivery of its main components - the Cas9 endonuclease and a guide RNA - into cells. Here, we describe the development of a novel transient and non-viral Cas9 delivery strategy based on the translocation machinery of the *Bacillus anthracis* anthrax toxin, PA (protective antigen). We show that Cas9 variants fused to the N-terminus of the lethal factor or to a hexahistidine tag are shuttled through channels formed by PA into the cytosol of human cells. As proof-of-principle, we applied our new approach, denoted as CRISPA, to knock out lipolysis-stimulated lipoprotein receptor (LSR) in the human colon cancer cell line HCT116 and green-fluorescent protein (GFP) in human embryonic kidney 293T cells stably expressing GFP. Notably, we confirmed that the transporter PA can be adapted to recognize specific host cell-surface receptor proteins and may be optimized for cell type-selective delivery of Cas9. Altogether, CRISPA provides a novel, transient and non-viral way to deliver Cas9 into specific cells. Thus, this system is an additional step towards safe translation of the CRISPR/Cas9 technology into clinics.

## Introduction

CRISPR/Cas9 emerged over the last years as a key technology for targeted genome editing. The cooperative assembly of the Cas9 endonuclease and a target gene-specific guide RNA (gRNA) allows the introduction of a site-specific double-strand-break (DSB). Repair of the DSB via error-prone non-homologous end joining (NHEJ) eventually leads to frequent base pair insertions or deletions (indels), which disrupt gene expression (Mali et al., 2013). Conventionally, Cas9 and the gRNA sequence are transiently or stably introduced into cells via plasmid- or viral-based delivery methods. To this end a plethora of different delivery methods, including electroporation, cell penetrating peptides or as Cas9-gRNA ribonucleoproteins (RNPs), were described (Nelson and Gersbach, 2016; Liu et al., 2017; Lino et al., 2018; Wang et al., 2019). Most *in vivo* Cas9-based genome editing trials aimed to eradicate integrated E6 and E7 HPV (human papilloma virus) oncogenes in accessible cervical cancer. Cas9-guided therapy has been applied to the eye, e.g. for the repair of inherited retinal disorders (Hirakawa et al., 2020). However, the lack of cell-type/tissue selectivity remains a drawback and a major hurdle in translating CRISPR/Cas9 genome editing into clinical applications. Increasing cell specificity of CRISPR/Cas9 genome editing would allow to expand its therapeutic applications to a larger number of diseases, beyond easily accessible tissues.

A delivery system based on bacterial toxins can be engineered to become highly cell type-specific (Beilhartz et al., 2017). In principle, the receptor-binding domain of the toxin can be substituted with a protein ligand for a cell surface receptor that is expressed exclusively in a specific cell type. Anthrax lethal toxin, the main virulence factor of the anthrax-causing pathogen *Bacillus anthracis*, is a binary combination of a protein transporter system (PA) and the executor factor (lethal factor, LF). LF is a protease which specifically cleaves mitogen-activated protein kinases (MAPKs), ultimately leading to cell cycle arrest and an accompanying inhibition of cell proliferation (Moayeri and Leppla, 2009). PA acts as the binding and translocation component for the biologically active toxin complex. Upon binding to its target cell receptors ANTXR1 (TEM8) and ANTXR2 (CMG2) (van der Goot and Young, 2009), a 20 kD fragment of the 83 kD PA (PA83) is released by proteolytic cleavage. Processed PA (now PA63) forms heptamers or octamers at the cell surface and recruits up to three LF molecules to the PA63 oligomer/receptor complex. Following receptor-mediated endocytosis and acidification of endosomes by vesicular proton pumps (vATPases), PA63 oligomers form channels in endosomal membranes, facilitating the translocation of LF into the host cell cytosol (Young and Collier, 2007; Moayeri et al., 2015).

More than 2 decades ago, it was shown that foreign proteins can be delivered into mammalian cells via PA, when fused to the PA recognition domain of LF (N-terminus, LF_N_) (Arora and Leppla, 1993, Arora and Leppla, 1994; Milne et al., 1995). PA-dependent delivery to the cytosol is also facilitated when a short polybasic sequence of lysine, arginine or histidine residues is fused to cargo proteins (Blanke et al., 1996; Sharma and Collier, 2014). Importantly, a conventional, N-terminal hexahistidine tag (His6-tag), which is commonly used as a purification tag for recombinant proteins, is also sufficient for promoting cell entry of foreign proteins into cells via the PA channel (Beitzinger et al., 2012). To date more than 30 different non-native cargos have been delivered into the cytosol of cells by using PA as non-toxic delivery platform to probe intracellular biological functions (Rabideau and Pentelute, 2016; Piot et al., 2021).

In the present study, the PA-mediated delivery platform was established as a non-viral, transient Cas9 system for RNA-guided CRISPR/Cas9 genome engineering. As proof-of-concept, we generated a knockout of the gene encoding the protein LSR (lipolysis-stimulated lipoprotein receptor) in the human colon carcinoma cell line HCT116, since we previously succeeded in knocking out the lsr gene with the conventional CRISPR/Cas9 mutagenesis approach in this cell line (Hemmasi et al., 2015). LSR serves as host entry receptor for the AB-type binary actin-ADP-ribosylating *Clostridium difficile* toxin CDT (*Clostridium difficile* transferase) (Papatheodorou et al., 2011). LSR-deficient HCT116 cell clones are CDT-resistant and can easily be selected after toxin treatment. In addition, the system was translated to a different setting by targeting stably integrated GFP in the commonly used human embryonic kidney cell line 293T. Finally, we demonstrate cell specificity with a mutated PA variant fused to a growth factor, which enabled the specific transport of a toxic cargo protein into cells expressing the corresponding growth factor receptor.

This novel modular PA-based Cas9 delivery method, denoted as CRISPA (a portmanteau of CRISPR and PA), represents an innovative tool in molecular biology with future clinical potential especially for *ex vivo* gene therapy purposes. Moreover, CRISPA can be further developed for targeted, cell type-selective delivery of Cas9 by changing the receptor-specificity of PA.

## Materials and Methods

### Cell Culture and Transfection

HEK293T cells (ATCC: #CRL-3216) were cultured in Dulbecco’s Modified Eagle’s Medium (DMEM) supplemented with 10% (v/v) fetal calf serum (FCS), 1% (v/v) penicillin/streptomycin, and 2 mM L-glutamine. Wild-type HCT116 (ATCC: #CCL-247) and LSR-deficient HCT116/ΔLSR_CRISPR_ cells (Hemmasi et al., 2015) were cultivated in DMEM supplemented with 10% (v/v) FCS, 1% (v/v) penicillin/streptomycin, and 2 mM L-glutamine. HeLa cells were grown in Minimum Essential Medium (MEM) supplemented with 10% (v/v) FCS, 1% (v/v) penicillin/streptomycin, 2 mM L-glutamine, and 1 mM sodium pyruvate. A431 cells were grown in Roswell Park Memorial Institute (RPMI) 1640 Medium supplemented with 10% (v/v) FCS, 1% (v/v) penicillin/streptomycin, and 2 mM L-glutamine.

For HCT116 cells, Lipofectamine 2000 Transfection Reagent (Thermo Fisher Scientific, Waltham, United States) and for 293T-GFP cells, TransIT-LT1 Transfection Reagent (Mirus Bio LLC, Madison, United States) were used for transfection, according to the manufacturer’s protocols.

### Cell Viability Assay

Cell viability measurements with cultured cells were performed with the CellTiter 96 AQ_ueous_ One Solution Cell Proliferation Assay (MTS) from Promega (Madison, United States), according to the manufacturer’s protocol. Briefly, cells were seeded in 96-well plates and the next day the medium (100 µL) was exchanged with medium (100 µL) containing the respective toxin components at indicated concentrations. Mock- and DMSO (10% (v/v))-treated cells served as positive and negative control for cell viability, respectively. Following an incubation period of 24 h at 37°C, MTS (3-(4,5-dimethylthiazol-2-yl)-5-(3-carboxymethoxyphenyl)-2-(4-sulfophenyl)-2H-tetrazolium) was added to the medium of each well and the absorption was measured at 490 nm using a microtiter plate reader.

### Limited Dilution Cloning

Monoclonal cell populations were obtained by limited dilution cloning. Briefly, suspensions of trypsinized cells were adjusted with growth medium to a cell density of 5 cells per ml and transferred to 96-well plates in 100 µL portions (corresponding to 0.5 cells per well). An inverted microscope Axiovert ProgRess C10 plus was used to detect wells that contained only one defined cell clone after 7 days of growth. Single cell colonies were then removed by trypsinization and transferred to culture dishes for expansion.

### Generation of HEK293T Cells Stably Expressing GFP

GFP expressing HEK293T cells were generated with third generation lentiviral particles (Dull et al., 1998) as described previously (Koepke et al., 2020). In short, third generation lentiviruses were produced using pCSC-SP-PW-GFP and helper plasmids and then used to transduce HEK293T cells. Three days post transduction, the cells were sorted using the BD FACSAria II. Cells with moderate GFP MFIs were pooled and cultivated at 37°C with 5% (v/v) CO_2_ under humidified conditions. The pCSC-SP-PW-GFP plasmid (aka pBOB-eGFP) was kindly provided by Inder Verma (Addgene, #12337 (Marr et al., 2004)).

### Recombinant Toxins or Toxin Components

Recombinantly expressed and purified toxins or toxin components, such as His-TccC3hvr, C2I, C2II, CDTa, CDTb, PA (PA83) and the receptor-binding domain (RBD) of CDTb, are described elsewhere (Barth et al., 2000; Tonello et al., 2004; Lang et al., 2010; Schwan et al., 2011; Papatheodorou et al., 2013). The RBD of CDTb was used as a glutathione S-transferase (GST) fusion protein. The binding components of CDT, C2 and anthrax toxin, namely CDTb, C2II and PA83, respectively, were used as protease-activated proteins, whereas protease-activated PA83 is denoted as PA63 (Singh et al., 1989; Barth et al., 2000; Blöcker et al., 2001; Sterthoff et al., 2010). Unnicked diphtheria toxin (DT) from *Corynebacterium diphtheriae* was purchased from Sigma-Aldrich (order number: D0564). His-TccC3hvr, both CDT components (CDTa and CDTb) and the RBD of CDTb were provided by the laboratory of Klaus Aktories (University of Freiburg, Germany). The PA variants mPA83 and mPA83-EGF as well as LF_N_-DTA were provided by R. John Collier (Harvard Medical School, Boston, United States).

Subconfluent monolayers of cultured cells were intoxicated by direct addition of toxins or toxin components into the growth medium. If required, toxin dilutions were performed with growth medium or phosphate-buffered saline. If not otherwise stated, the final concentrations of the toxins during the intoxication were as follows: CDTa/CDTb (5 nM/1 nM), C2I/C2II (1 nM, 1.67 nM), PA63/His-TccC3hvr (10 nM/50 nM), DT (10 nM), and the combinations PA83, PA63, mPA83 or mPA83-EGF (20 nM) plus LF_N_-DTA (10 nM), respectively. The intoxication of cells was analyzed by microscopic evaluation of the cell morphology, by measuring cell viability or by immunodetection of target substrate modification.

### Generation, Expression and Purification of His- and LF_N_-Cas9

For the recombinant production of His-Cas9, *Escherichia coli* XJb (DE3) Autolysis strain (T3051, Zymo Research Europe, Freiburg, Germany) was transformed with Addgene plasmid #53261 (pET28a/Cas9-Cys (Ramakrishna et al., 2014)). Briefly, transformants were grown in liquid LB medium at 37°C until an OD_600 nm_ of 0.6–0.8 was reached. Then, 0.5 mM IPTG and 3 mM arabinose were added to the medium for His-Cas9 and lambda lysozyme expression, respectively, followed by incubation for 4 h at 37°C under shaking. After harvesting of the cells by centrifugation, cells were resuspended in buffer, containing 50 mM NaH_2_PO_4_ (pH 8), 300 mM NaCl, 5 mM imidazole, 0.03% (v/v) Triton X-100, and stored at −20°C. A cell lysate for subsequent protein purification was generated by thawing the cell pellet, followed by addition of 1 mM PMSF and 1 mM DTT to the cell suspension. Next, cell debris was removed by centrifugation (30,000 g, 30 min, 4°C) and the supernatant filtered through a 0.45 and 0.2 µm sterile filter. Finally, the filtrate was subjected to PureCube 1-step batch Midi Plus Columns (Cube Biotech, Monheim, Germany) for purification of His-Cas9 by nickel affinity chromatography. Washing and elution buffers were 50 mM NaH_2_PO_4_ (pH 8), 300 mM NaCl, 20 mM imidazole, 0.03% (v/v) Triton X-100 and 50 mM NaH_2_PO_4_ (pH 8), 300 mM NaCl, 250 mM imidazole, respectively. Eventually, a buffer exchange of the eluate to phosphate-buffered saline (PBS) was performed with Vivaspin 20 columns (Sigma-Aldrich Chemie GmbH, Munich, Germany).

LF_N_-Cas9 was expressed and purified essentially as described for His-Cas9 above. However, the initial cloning of the LF_N_-Cas9 fusion construct was necessary. The Addgene plasmid #62933 (pET-Cas9-NLS-6xHis (Zuris et al., 2015)) served as backbone for insertion of an LF_N_-encoding fragment upstream of the Cas9 sequence by seamless assembly (NEBuilder HiFi DNA Assembly Cloning Kit; #E5520; New England BioLabs GmbH, Frankfurt am Main, Germany). The LF_N_-encoding fragment was generated by PCR and with Addgene plasmid #11075 (pET-15b LFN-DTA (Milne et al., 1995)) as template. The NEBuilder Assembly Tool (New England BioLabs GmbH, Frankfurt am Main, Germany) was used to design primers for the seamless cloning procedure.

Purified His- and LF_N_-Cas9 proteins were tested for dsDNA cleavage activity by performing an *in vitro* Cas9 cleavage assay with the CRISPRcraft S. p. Cas9 Control Kit (Lucigen, Middleton, United States), which includes a substrate DNA, a substrate DNA-specific gRNA and a set of DNA primers. For the *in vitro* cleavage assay, ∼300 ng of a 1083 bp DNA fragment (generated by PCR and by using the supplied substrate DNA as template) was incubated for 10 min together with the gRNA and 20 nM or 40 nM His-Cas9- and LF_N_-Cas9, respectively, and cleavage products were analyzed by agarose gel electrophoresis. The gRNA-directed cleavage of the 1083 bp fragment results into two fragments with the size of 800 and 283 bp, respectively. Commercial Cas9 protein (EnGen^®^ Spy Cas9 NLS from New England Biolabs (NEB); M0646T) was used as positive control for *in vitro* DNA cleavage.

For the detection of His- and LF_N_-Cas9 via immunoblotting and enhanced chemiluminescence (ECL) imaging, a mouse anti-His (AD1.1.10) antibody (Santa Cruz Biotechnology, Dallas, United States) as primary antibody in combination with mouse IgGκ light chain binding protein coupled to horseradish peroxidase (m-IgGκ BP-HRP) was used.

### Immunodetection of Proteins in Whole-Cell Lysates

Whole-cell lysates were generated directly in well plates or culture dishes by washing cell monolayers first with PBS and then by resuspending them in Laemmli buffer. Prior to SDS-PAGE followed by immunoblotting, lysate samples were heated at 95°C for 5 min. For the detection of LSR, Hsp90 and GAPDH, commercial primary antibodies (rabbit anti-LSR (X-25); mouse anti-Hsp90 α/β (F-8); mouse anti-GAPDH (G-9)) and secondary antibodies (m-IgGκ BP-HRP and goat anti-rabbit IgG-HRP) were used. All antibodies were purchased from Santa Cruz Biotechnology, Dallas, United States.

### Light Microscopy

An inverted Axiovert 40 CFL microscope (Carl Zeiss Microscopy, Jena, Germany) equipped with a ProgRess C10 plus camera (Jenoptik, Jena, Germany) was used in this study for light microscopic analyses, such as monitoring cell intoxication, identification of growing cell clones in wells and cell confluence estimation, respectively.

### Verification of Cas9-Induced Indel Mutations in LSR Knockout Clones

First, genomic DNA was isolated from 1 × 10^6^ cells using the MyTaq Extract-PCR Kit (Bioline GmbH, Luckenwalde, Germany) or innuPREP DNA Mini Kit (Analytik Jena, Jena, Germany) following the manufacturer’s recommendations. Next, a 403 bp DNA fragment within the lsr gene that contains the LSR-specific gRNA binding site was amplified by PCR with oligonucleotides 5′-GTC​CAA​CCC​CTA​CCA​CGT​GGT​G-3′ and 5′- GCT​TTC​AGA​TGG​GGA​CTC​CAG​G-3′ and by using genomic DNA as template (Hemmasi et al., 2015). Eventually, the PCR product was purified with the my-Budget Double Pure Kit (BioBudget Technologies GmbH, Krefeld, Germany) and subjected to DNA sequencing (Eurofins Genomics Europe Sequencing GmbH, Konstanz, Germany). Indels in the sequenced DNA fragments were identified by performing sequence alignments with the lsr reference sequence.

### Sequential ADP-Ribosylation

The sequential ADP-ribosylation assay served to estimate *in vitro* the ADP-ribosylation status of cellular actin after intoxication of cells by the actin ADP-ribosylating toxin CDT (Kaiser et al., 2011). First, intoxicated cells (and mock-treated cells as negative controls) were washed twice with PBS and frozen at −20°C. Then, cell lysis was induced by thawing, after which the lysed cells were collected with a cell scraper and resuspended in buffer containing 20 mM Tris-HCl (pH 7.5), 5 mM MgCl_2_, 1 mM EDTA, 1 mM DTT, and cOmplete protease Inhibitor Cocktail (Roche, Rotkreuz, Switzerland). Cell lysate (20 µL) was incubated for 30 min at 4°C together with purified CDTa (100 ng) and 250 pmol biotinylated NAD^+^ (Trevigen, Gaithersburg, United States), prior to addition of Laemmli buffer and heating for 10 min at 95°C to stop the ADP-ribosylation reaction. Eventually, *in vitro* sequentially ADP-ribosylated (and thus biotinylated) actin was visualized by Western Blotting and ECL and by the use of peroxidase-conjugated streptavidin (Merck, Darmstadt, Germany).

### Flow Cytometry

For the analysis of cell surface binding, purified proteins were fluorescently labelled with DyLight 488 NHS Ester (Thermo Fisher Scientific, Waltheim, United States) following manufacturer’s instructions and as described earlier (Papatheodorou et al., 2013). Zebra Spin Desalting Columns (Thermo Fisher Scientific, Waltham, United States) were used to remove excess fluorescent dye after the labelling reaction. Labelling efficiency and protein concentration were determined by performing spectrophotometric analyses on a NanoDrop OneC spectrophotometer (Thermo Fisher Scientific, Waltham, United States). Next, cultured cell monolayers were detached by incubation for 15–30 min at 37°C with PBS containing 25 mM EDTA (and without trypsin) to preserve proteins at the cell surface. The detached cells were resuspended in growth medium and the cell density of the cell suspension adjusted to 1 × 10^6^ cells per ml. Then, DyLight-labelled protein at indicated amounts was incubated with 2 × 10^5^ cells (200 μL cell suspension) for 30 min on ice to prevent cellular uptake of cell surface-bound proteins. Finally, cells were washed up to 5 times by centrifugation (500 g, 5 min, 4°C) and resuspension in PBS to remove any non-bound DyLight-labelled protein, prior to flow cytometric analysis of the cell suspensions using the BD FACSCelesta flow cytometer run by the FACSDiva software (BD Biosciences, San Jose, United States). The results of the flow cytometric measurements (typically 1 × 10^4^ cells per sample) were further analyzed using Flowing software version 2.5.1 (University of Turku, Finland).

The sorting of HEK293T cells expressing moderate GFP levels was done using the BD FACSAria III. To measure GFP fluorescence of cells applied to the CRISPA method the Beckman-Coulter CytoFLEX was used.

### Guide RNA Expression Plasmids

Guide RNA (gRNA) expression plasmids including the protospacer sequences 5′- GGA​CAG​CGT​GCG​CAC​CGT​CA-3 for LSR and 5′-GTG​AAC​CGC​ATC​GAG​CTG​AA-3′ for GFP were used in this study that are described elsewhere (Hemmasi et al., 2015; Koepke et al., 2020).

### T7EI Mismatch Detection Assay

293T-GFP cells were cultivated in a 24-well to half-confluency, then transfected with a plasmid encoding a GFP-specific gRNA, followed by medium renewal after 6 h. The next day, the cells were incubated for 24 h with 10 nM PA63 together with 100 nM His- and LF_N_-Cas9, respectively, or a commercially available His-Cas9 protein (EnGen^®^ Spy Cas9 NLS from New England Biolabs (NEB); M0646T). Cells were then harvested for T7 endonuclease I (T7EI) mismatch detection using the GeneArt^®^ Genomic Cleavage Detection Kit (Thermo Scientific). In short, total genomic DNA was extracted from harvested cells and the locus, which is targeted by the GFP-specific gRNA was amplified by PCR using the primers 5′-ACA​GCT​CGT​CCA​TGC-’3 (GFP-rev) and 5′-TGC​TTC​AGC​CGC​TAC​C-’3 (GFP-fwd). Subsequently, the PCR product was heat-denatured and reannealed to generate mismatch strands. These mismatches were then detected and cleaved by the Detection Enzyme (T7EI) and the resulting products were separated by agarose gel electrophoresis and visualized by gel imaging (Gel Doc XR + Gel Documentation System; Biorad). DNA band intensity was analyzed with ImageJ (Schneider et al., 2012) and the cleavage efficiency calculated essentially as described in the GeneArt^®^ Genomic Cleavage Detection Kit manual.

### Statistics

Bar diagrams were generated with mean values calculated from triplicates or from three samples performed in parallel and with error bars representing standard deviation (SD). Significance differences were calculated with Microsoft Excel (Student´s) *t*-test. Resulting *p* values were indicated by asterisks as follows: *p < 0.05, **p < 0.01, ***p < 0.001.

## Results

### Establishing CRISPA in HCT116 Cells

**FIGURE 1:**
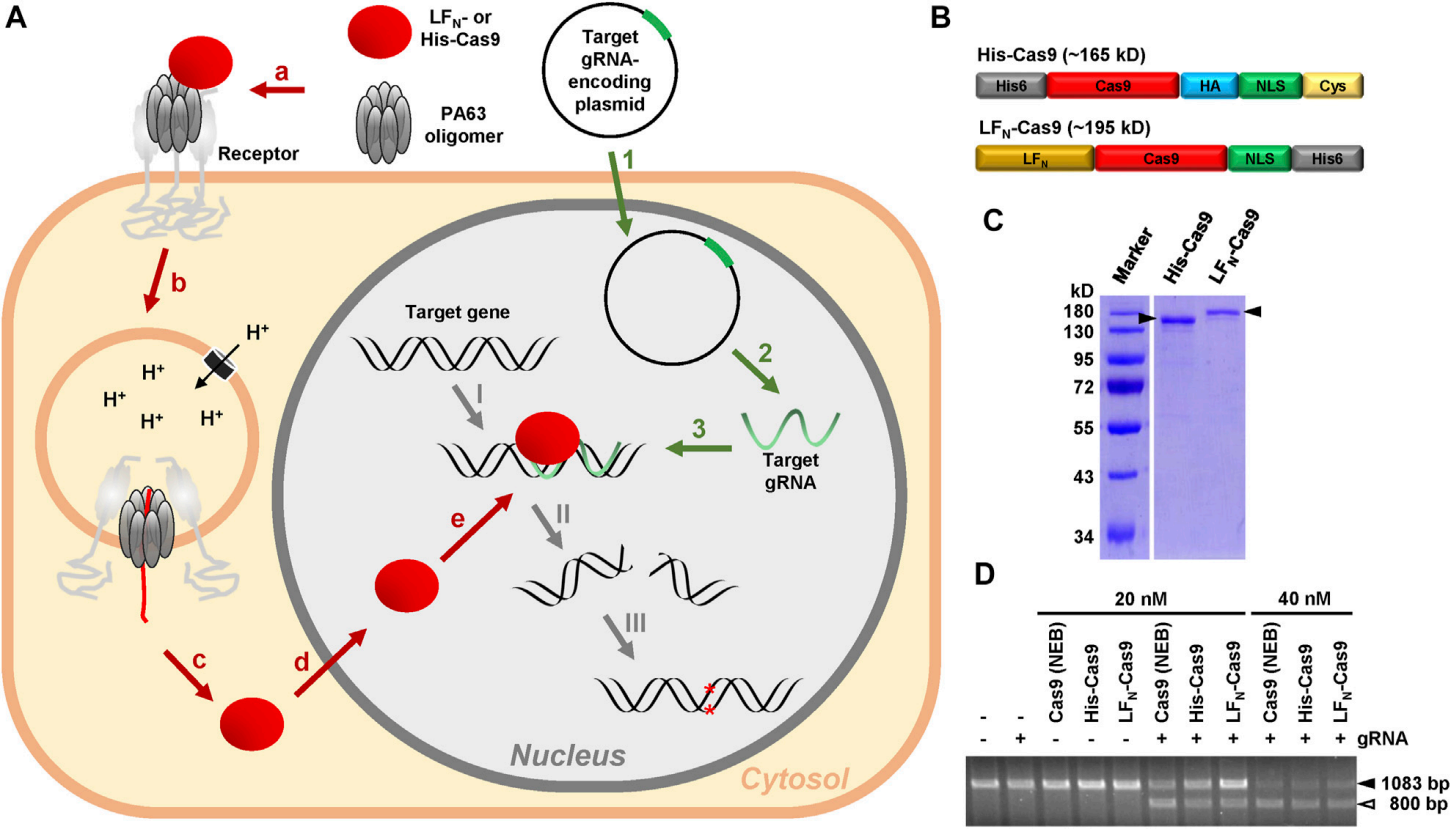
Rationale of the modular CRISPA concept and characterization of His- and LF_N_-Cas9. **(A)** 1: A plasmid for the expression of the target gRNA is introduced into cells via transfection; 2: Target gRNA is expressed by the cells; 3: Target gRNA forms a RNA-DNA-heteroduplex at its target position within the genomic DNA; a: PA63 binds as oligomer to its receptor on the cell surface and forms a complex with LF_N_- or His-Cas9; b: Receptor-mediated endocytosis promotes entry of the PA63 oligomer–LF_N_-/His-Cas9–receptor complex into cells via endosomes; c: Acidification of the endosomes leads to channel formation of the PA63 oligomer into the endosomal membrane and translocation of unfolded LF_N_-/His-Cas9 into the cytosol; d: The nuclear localization signal (NLS) promotes entry of refolded LF_N_-/His-Cas9 into the nucleus; e: LF_N_-/His-Cas9 is recruited to the RNA-DNA-heteroduplex formed by the gRNA at its target position: I: A LF_N_-/His-Cas9–gRNA complex is formed at the target gene. II: LF_N_-/His-Cas9 leads to a double-strands cleavage of the target gene; III: Non-homologous end joining (NHEJ) generates indels into the target gene, which in turn lead to premature stop codons within the open reading frame (ORF) of the targeted gene, resulting in the knockout of the respective gene **(B)** Schematic representation of the modular composition of His-Cas9 (∼165 kD) and LF_N_-Cas9 (∼195 kD). Please note that the size of the boxes is not related to the size of the modules. His6, hexahistidine tag; HA, human influenza hemagglutinin epitope tag; NLS, nuclear localization sequence; Cys, cysteine residue **(C)** Coomassie-stained polyacrylamide gel for size and purity control of purified His- (0.55 µg) and LF_N_-Cas9 (0.425 µg) indicated by filled arrowheads **(D)** Cas9 cleavage assay. Cleavage of approx. 300 ng target DNA (1083 bp; filled arrowhead) after incubation with 20 nM or 40 nM His- or LF_N_-Cas9, respectively, or commercial Cas9 (Cas9 (NEB)) alone or in combination with the respective guide RNA (gRNA) was monitored by agarose gel electrophoresis. The open arrowhead indicates the 800 bp cleavage product of the target DNA. bp, base pairs.

Here, we describe a novel modular PA-based Cas9 delivery concept, denoted as CRISPA, that can be subdivided into three main processes. The first is the transfection of a plasmid encoding the target guide RNA into cells. The second step is the delivery of Cas9 into cells via PA. As a consequence of both processes, a third process is initiated, the Cas9-dependent cleavage of the target gene, eventually resulting in the knockout of the respective gene (Figure 1A).

The CRISPA method requires purified recombinant Cas9 protein, either fused to an N-terminal His6-tag (denoted as His-Cas9) or to the N-terminus of the *Bacillus anthracis* lethal factor (denoted as LF_N_-Cas9). Suitable Cas9 constructs must also possess a nuclear localization signal (NLS), in order to reach the nucleus of target cells. As His-Cas9, we used a recently described Cas9 construct consisting of an N-terminal His6-tag and Cas9 followed by an HA tag, an NLS and a single cysteine residue (Ramakrishna et al., 2014). As LF_N_-Cas9, a Cas9 construct consisting of Cas9 followed by an NLS and a His6-tag was used, where we fused the N-terminus of the lethal factor (LF_N_) at its N-terminus by Gibson cloning (Zuris et al., 2015) (Figure 1B). Both Cas9 fusion proteins were expressed in *Escherichia coli* and purified as His-tagged proteins by nickel affinity chromatography. The purity of both Cas9 preparations (∼87% for His- and 82% for LF_N_-Cas9) was determined by SDS-PAGE and Coomassie staining (Figure 1C). Both Cas9 preparations were almost as active as commercial Cas9 protein in an *in vitro* Cas9 cleavage assay (Figure 1D).

We have chosen the HCT116 cell line as a model cell line for our CRISPA approach, because we previously succeeded in knocking out a gene of interest via the conventional CRISPR/Cas9 approach in these cells (Hemmasi et al., 2015). To examine whether HCT116 cells are a suitable target for the CRISPA approach, we first determined whether PA (in particular PA63 was applied in this study) is able to promote translocation of foreign cargo proteins into HCT116 cells. Here, we used His-tagged TccC3hvr (actin ADP-ribosyltransferase domain of the *Photorhabdus luminescens* PTC3 toxin) as toxic cargo protein for PA, since it was reported that this combination is toxic to various mammalian cell lines, such as HeLa and Vero (Lang et al., 2010; Ernst et al., 2017). Beitzinger et al. utilized 3 μg/ml (∼50 nM) proteolytically-activated PA (PA63) for transport of a His- or LF_N_-fused cargo protein into human umbilical vein endothelial cells (HUVECs) (Beitzinger et al., 2012). We tested whether 10 nM PA63 would already be sufficient for translocating His-TccC3hvr at three different concentrations (20, 50, 100 nM) into HCT116 cells.

Mock-treated HCT116 cells or HCT116 cells treated for up to 24 h with 100 nM His-TccC3hvr or 10 nM PA63 alone did not show any morphological signs of intoxication (Supplementary Figure S1A). However, HCT116 cells incubated with the combination of 10 nM PA63 plus 20, 50 or 100 nM His-TccC3hvr, displayed strong cell rounding, a typical hallmark of TccC3hvr intoxication due to its molecular action on the actin cytoskeleton, already after 6 h of incubation (Supplementary Figure S1B and S1C). Thus, 10 nM PA63 is sufficient for translocating His- or LF_N_-fused cargo proteins into HCT116 cells.

These results indicated that HCT116 cells express host receptor(s) for PA and permit transport of its cargo protein.

### Generation of HCT116 LSR Knockout Cells via CRISPA

**FIGURE 2:**
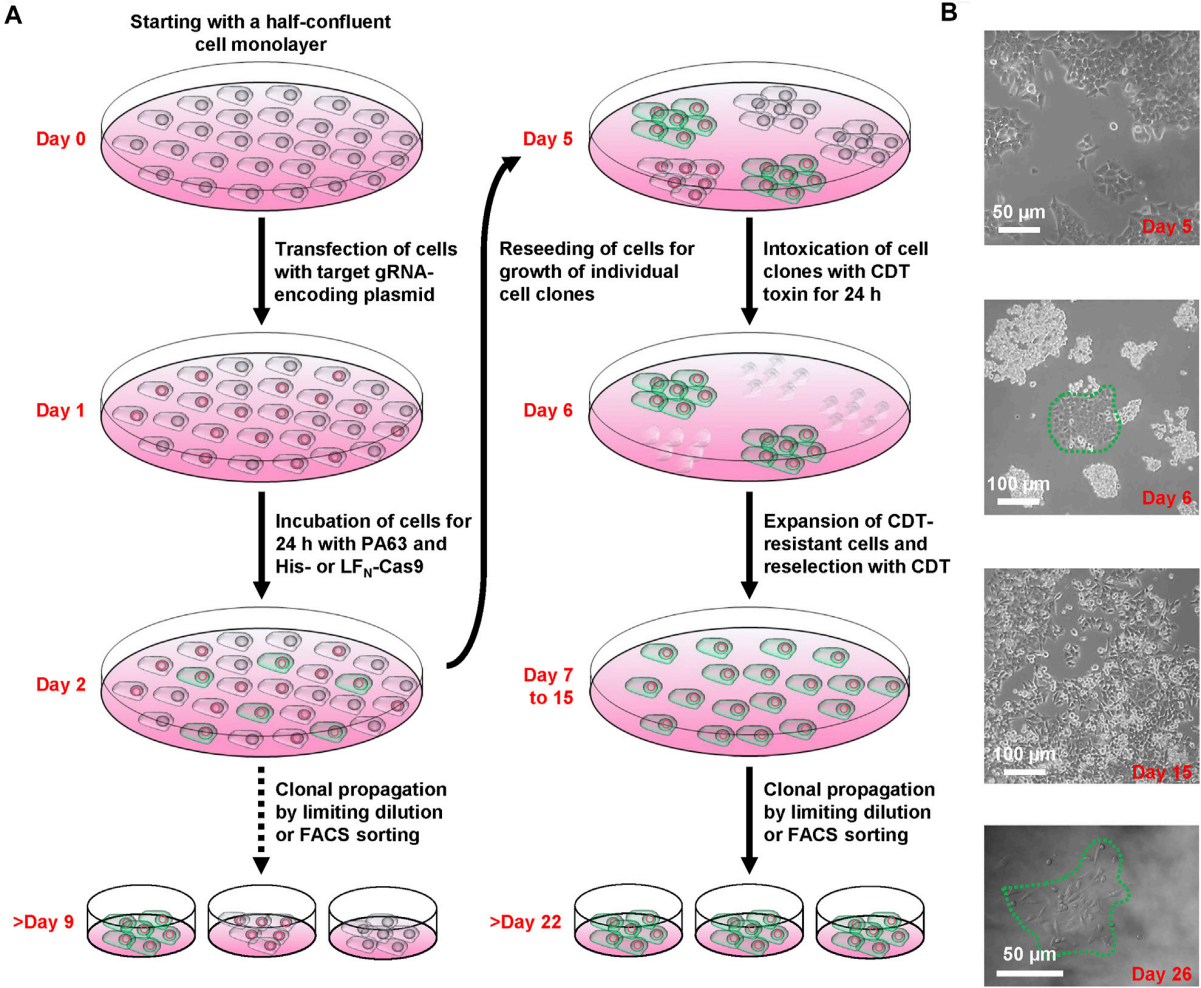
Working scheme of the CRISPA mutagenesis protocol. **(A)** Illustration of the successive steps of the CRISPA approach with images of key steps shown in **(B)**.

We next set about knocking out LSR in HCT116 cells by applying the CRISPA method (Figure 2A). At first, HCT116 cells were cultivated in a 24-well plate to half-confluency (day 0), then transiently transfected with a plasmid encoding the LSR-specific gRNA and the medium renewed 6 h after transfection. At day 1, cells were treated for 24 h with 10 nM PA63 together with 100 nM His-Cas9 or LF_N_-Cas9, respectively, to induce the Cas9-based knockout of LSR. It is of note that already at this stage (day 2), single cell clones for further analysis and identification of cell clones carrying the desired gene knockout may be isolated.

LSR is the host entry receptor for CDT. This enabled us to further select for LSR-deficient HCT116 cells simply by intoxicating the cells after CRISPA with CDT. To this end, cells were trypsinized at day 2 and one-tenth of the cells was re-seeded into a 6-well to obtain segregated colonies of clonal derivatives at the plastic bottom. Grown cell colonies were then incubated twice for 24 h with CDT at day 5 and day 6. At day 7, CDT-resistant cell clones could easily be discriminated microscopically from non-resistant cell clones that exhibited massive CDT-induced cell rounding. Only a few CDT-resistant cell clones were observed in each 6-well (typically up to 10), independent of whether His- or LF_N_-Cas9 was used together with PA63. Notably, CDT-resistant cell clones were only detected when all components of the CRISPA approach (LSR-specific guide RNA-expressing plasmid, PA63 and His-/LF_N_-Cas9) were used in combination, indicating that CDT-resistant cell clones did not arise from random mutations due to genetic instability. CDT-resistant cells were expanded and reselected again with CDT (days 7–15, Figure 2B). Eventually, single cell clones were obtained in 96-wells by limiting dilution and cell colonies were clearly visible after approximately 1 week of incubation (>day 22) (Figure 2B).

### Verification of the LSR Knockout in HCT116 Cell Clones

**FIGURE 3:**
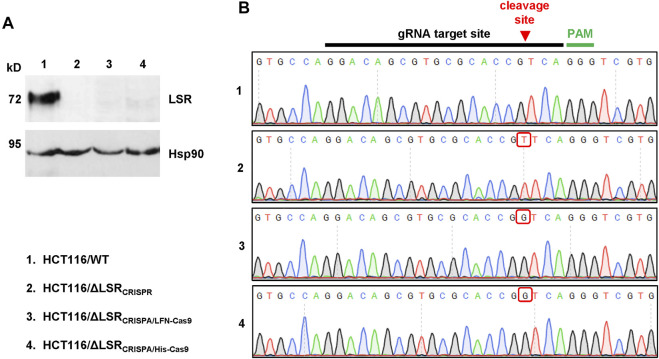
Confirmation of the CRISPA-mediated LSR knockout mutation in HCT116 cells. **(A)** Immunoblot for the detection of LSR and Hsp90 (loading control) in whole-cell lysates of wildtype and LSR-deficient HCT116 cells (a detailed description of the cells is provided in the legend of numbers within the figure) **(B)** Chromatograms obtained by Sanger sequencing are shown, comprising the gRNA target site, the Cas9 cleavage site (red arrow) and the PAM sequence with the LSR gene of wildtype and LSR-deficient HCT116 cells (a detailed description of the cells is provided in the legend of numbers within the figure). Red box points towards a nucleotide insertion (mutation) that has been generated as a result of Cas9 cleavage and non-homologous end joining.

To test whether the CRISPA approach was successful, we analyzed a single LF_N_-Cas9- (HCT116/ΔLSR_CRISPA/LFN-Cas9_) and a single His-Cas9-derived cell clone (HCT116/ΔLSR_CRISPA/His-Cas9_), respectively, via LSR immunoblotting. To this end, whole-cell lysates were generated from both CRISPA-derived LSR knockout cell clones and, for direct comparison, from wildtype HCT116 cells (HCT116/WT). Whole-cell lysates from LSR-deficient HCT116 cells generated using conventional CRISPR/Cas9 approach (denoted here as HCT116/ΔLSR_CRISPR_) (Hemmasi et al., 2015) were used as negative controls for LSR immunodetection. LSR immunoblotting confirmed the lack of LSR expression in HCT116/ΔLSR_CRISPR_, but also in HCT116/ΔLSR_CRISPA/LFN-Cas9_ and HCT116/ΔLSR_CRISPA/His-Cas9_ cells (Figure 3A).

Next, we analyzed whether indel mutations in our target gene LSR are present in both CRISPA-derived LSR knockout clones. For that purpose, genomic DNA was isolated from each cell clone and from HCT116/WT and HCT116/ΔLSR_CRISPR_ cells and a region including the guide RNA target site of the LSR gene was amplified by PCR and sequenced. In comparison to HCT116 wildtype cells, both the LF_N_-Cas9- and the His-Cas9-derived cell clone exhibited a frameshift mutation (single-guanine insertion) directly within the Cas9 cleavage site of the LSR guide RNA target site. A single-thymine insertion was found in HCT116/ΔLSR_CRISPR_ cells at this position (Figure 3B). Both mutations lead to premature stop codons within the open reading frame (ORF) of LSR, resulting in a loss of expression of LSR.

**FIGURE 4:**
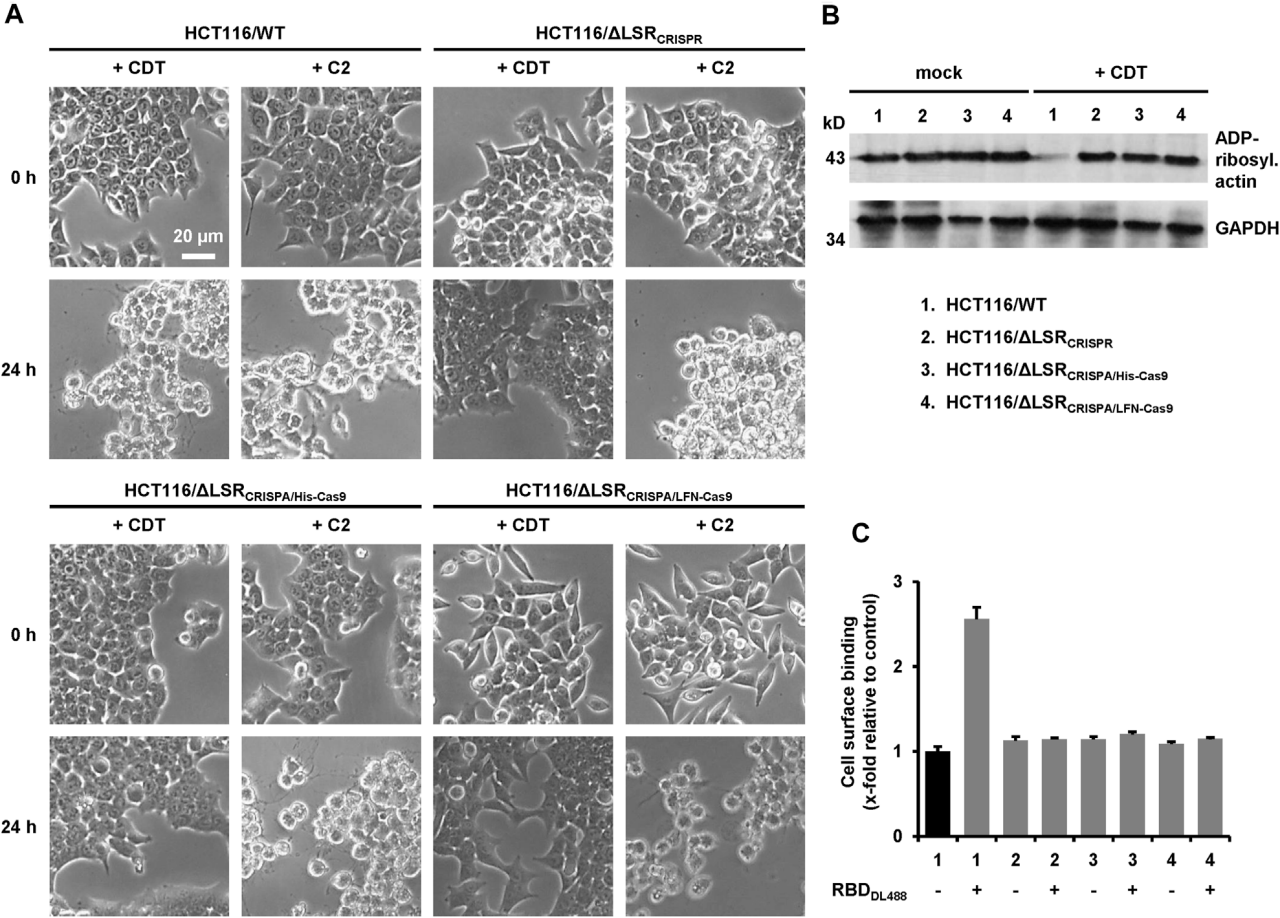
Characterization of LSR-deficient HCT116 cells with CDT. **(A)** Wildtype HCT116 cells (HCT116/WT) as wells as LSR-deficient HCT116/ΔLSR_CRISPR_, HCT116/ΔLSR_CRISPA/His-Cas9_ and HCT116/ΔLSR_CRISPA/LFN-Cas9_ cells were intoxicated with CDT (5 nM CDTa/1 nM CDTb) or C2 toxin (1 nM C2I/1.67 nM C2II) and images were obtained microscopically after 0 and 24 h to monitor morphological changes (cell rounding) of the cells. A scale bar representative for all images is shown in the upper left image **(B)** Wildtype and LSR-deficient HCT116 cells as described in the legend of numbers were intoxicated for 24 h with CDT (5 nM CDTa/1 nM CDTb) or were left untreated (mock), followed by a sequential ADP-ribosylation assay. Immunodetection of GAPDH served as loading control **(C)** Binding of DyLight 488-labeled CDTb-RBD (RBD_DL488_) on wildtype and LSR-deficient HCT116 cells as described in the legend of numbers in **(B)** was analyzed via FACS and fluorescence intensity (cell surface-binding) is shown in the bar diagram (*n* = 3, ±SD) as x-fold relative to control (black bar; mock-treated wildtype HCT116 cells), which was set to 1.

We then examined whether CRISPA-derived LSR knockout cell clones became resistant towards CDT intoxication, either (I) via microscopic analysis of cell morphology or (II) by probing the ADP-ribosylation status of actin. Here, the *Clostridium botulinum* C2 toxin served as a control, as it enters the target cells independent of LSR (Papatheodorou et al., 2011). Consequently, HCT116/ΔLSR_CRISPA/His-Cas9_ and HCT116/ΔLSR_CRISPA/LFN-Cas9_ cells were intoxicated for 24 h with CDT or C2 toxin, followed by microscopic analysis of the cell morphology. Wildtype and HCT116/ΔLSR_CRISPR_ cells served as controls in the intoxication assay. As expected, cell rounding induced by the cell cytoskeleton-disrupting activity of CDT was observed in wildtype HCT116 cells, while LSR-deficient HCT116/ΔLSR_CRISPR_, HCT116/ΔLSR_CRISPA/His-Cas9_ and HCT116/ΔLSR_CRISPA/LFN-Cas9_ cells were resistant to CDT (Figure 4A). All cells were readily intoxicated by C2 toxin (Figure 4A), indicating that general endocytic processes are still functional in these cells.

To substantiate our finding that HCT116/ΔLSR_CRISPA/His-Cas9_ and HCT116/ΔLSR_CRISPA/LFN-Cas9_ cells are resistant to CDT intoxication, we probed the ADP-ribosylation state of actin in lysates of CDT-treated and non-treated HCT116/ΔLSR_CRISPA/His-Cas9_ and HCT116/ΔLSR_CRISPA/LFN-Cas9_ cells using an *in vitro* actin-ADP-ribosylation assay. Importantly, the amount of actin amendable to sequential ADP-ribosylation upon CDT intoxication was only decreased in HCT116 wildtype but not in LSR-deficient HCT116/ΔLSR_CRISPR_, HCT116/ΔLSR_CRISPA/His-Cas9_ and HCT116/ΔLSR_CRISPA/LFN-Cas9_ cells (Figure 4B).

To verify lack of the LSR receptor, we demonstrated that CDT is no longer able to bind to HCT116/ΔLSR_CRISPR_, HCT116/ΔLSR_CRISPA/His-Cas9_ and HCT116/ΔLSR_CRISPA/LFN-Cas9_ cells. CDT is an AB-type binary toxin, where its B (binding/translocation) component (CDTb) mediates binding to LSR via a C-terminal receptor-binding domain (RBD) (Papatheodorou et al., 2013). Thus, the RBD of CDTb was labelled with the fluorescent dye DyLight 488 and its binding to suspension cells from wildtype and LSR-deficient HCT116/ΔLSR_CRISPR_, HCT116/ΔLSR_CRISPA/His-Cas9_ and HCT116/ΔLSR_CRISPA/LFN-Cas9_ cells at 4°C (no endocytic uptake) was analyzed by flow cytometry. Only wildtype HCT116 cells exhibited an increase in cell surface-bound fluorescence after incubation with the fluorescent RBD, confirming that HCT116/ΔLSR_CRISPR_, HCT116/ΔLSR_CRISPA/His-Cas9_ and HCT116/ΔLSR_CRISPA/LFN-Cas9_ cells are lacking LSR expression at the cell surface (Figure 4C).

In summary, our results showed that the CRISPA method allowed knockout of a specific gene in a human cell line.

### Application of CRISPA to HEK293T Cells

**FIGURE 5:**
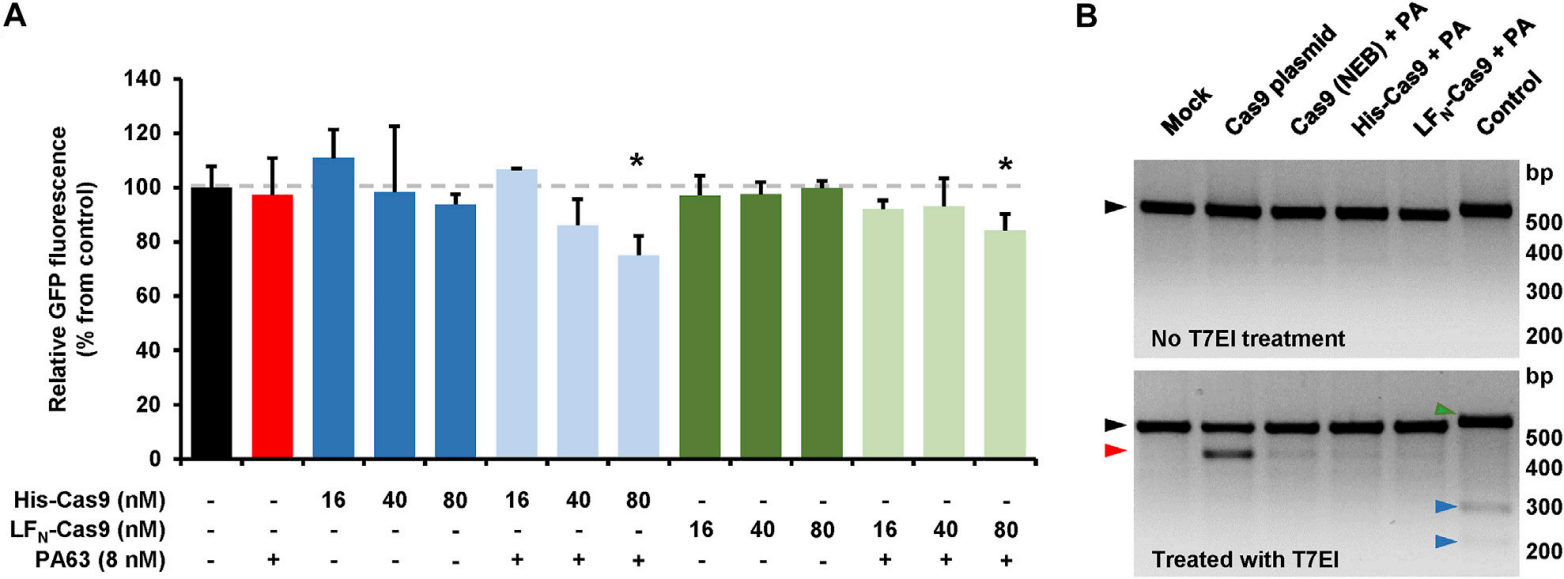
CRISPA-mediated knockout of GFP in 293T GFP cells. **(A)** 293T GFP cells transfected with a gRNA-expressing plasmid targeting GFP were incubated the following day either with indicated concentrations of His- or LF_N_-Cas9 alone (dark colored blue and green bars) or together with 8 nM PA63 (light colored blue and green bars). Red bar indicates cells treated with 8 nM PA63 only. Black bar represents mock-treated cells (control). Bar diagram shows the median GFP fluorescence of cells after 6 days of incubation relative to control, which was set to 100%. Results shown are mean values calculated from three samples performed in parallel and with error bars indicating *n* = 3 ± SD (*p < 0.05) **(B)** T7 endonuclease I mismatch detection assay. 293T-GFP cells transfected with a plasmid encoding a GFP-specific gRNA were incubated for 24 h with 10 nM PA63 (PA) together with 100 nM His- and LF_N_-Cas9, respectively, or a commercial Cas9 protein carrying an N-terminal His-tag (denoted as Cas9 (NEB)). Cells co-transfected with a Cas9-expressing plasmid (Cas9 plasmid) served as positive control and cells without Cas9 as negative control (mock). A T7 endonuclease I (T7EI) mismatch detection assay was performed with all cell populations and subsequently analyzed by agarose gel electrophoresis. Black triangles indicate PCR-derived DNA templates from all Cas9-treated cell populations with (lower panel) or without (upper panel) T7EI treatment. The red triangle indicates the T7EI-derived cleavage product in the Cas9-treated cell populations. Green and blue triangles indicate DNA template (516 bp) and cleavage products (225 and 291 bp) of the internal kit control (control), respectively. bp, base pairs **(A, B)** Experiments were performed at least twice with identical results.

We next sought to determine if the CRISPA approach is applicable in other cell lines and for different target genes. For that purpose, we used a HEK293T cell line stably expressing green-fluorescent protein (GFP). In a previous study it was already shown that HEK293T cells are susceptible to the lethal toxin of *B. anthracis* (Chavarría-Smith and Vance, 2013). Here, the target gene for the CRISPA approach was the non-native, chromosomally integrated coding sequence of GFP. Thus, GFP-expressing 293T cells (293T-GFP cells) transiently expressing a GFP-targeting gRNA were incubated with PA63 in combination with His- or LF_N_-Cas9, respectively. Cells were then incubated for 6 days, due to long half-life (∼26 h) of GFP in mammalian cells (Corish and Tyler-Smith, 1999) and GFP expression subsequently analyzed via flow cytometry. A clear decrease of the median fluorescence intensity (∼15–25%) of the 293T GFP cell population was observed, only if PA63 was added together with 80 nM His-Cas9 and LF_N_-Cas9, respectively (Figure 5A). Single treatment with either PA63 or any Cas9 variant did not affect GFP expression. This confirms cell entry of both Cas9 variants and consequently knockout of GFP via the PA transporter.

In order to estimate the genome editing efficiency in CRISPA-targeted 293T-GFP cells, a commercial kit-based T7 endonuclease 1 (T7EI) mismatch detection assay was performed. 293T-GFP cells transiently expressing a GFP-targeting gRNA were treated with PA63 and His- and LF_N_-Cas9. 293T-GFP cells co-transfected with a Cas9-expressing plasmid or treated with PA63 together with commercial Cas9 protein were used as controls. We observed genome editing efficiencies of 3.6/3.8% for His-Cas9, 2.4/2.6% for LF_N_-Cas9, 3.8/5.7% for commercial Cas9 (NEB), 20.4/20.5% for transfected Cas9 plasmid, and 7.8/8.8% for the internal control of the commercial kit (Figure 5B).

These results confirm that CRISPA approach can be applied to different cell lines and native or non-native target genes.

### Application of CRISPA to Selectively Target EGF Receptors Expressing Cells

The modular CRISPA approach has advantages over other non-viral and transient Cas9 delivery methods, because it offers the possibility for a cell-specific genome engineering. Previous studies have shown that PA can be engineered to bind to other host entry receptors. For instance, a mutated PA83 variant unable to bind to its native receptors due to mutations in the receptor-binding domain (termed mPA83), was fused to EGF (epidermal growth factor) and thus redirected to EGF receptors (EGFR), which are highly expressed in many cancer cells (Mechaly et al., 2012; Zahaf et al., 2017).

To confirm that mPA83-EGF selectively targets EGFR-positive cells, we used A431 and HeLa cells, which are characterized by high (A431 cells) and low (HeLa cells) levels of EGFR expression, respectively (Zhang et al., 2015). Both cell lines were intoxicated with 20 nM mPA83-EGF as the EGFR-specific transporter and 10 nM LF_N_-DTA as the toxic cargo component. In LF_N_-DTA, the N-terminus of the anthrax toxin lethal factor (LF_N_) is fused to the enzyme portion of the diphtheria toxin (DTA), which ADP-ribosylates the eukaryotic elongation factor 2 (eEF2) and thereby inhibits protein synthesis (Milne et al., 1995). Measuring the cell viability after 24 h of intoxication revealed that A431 (Supplementary Figure S2; black bars), but not HeLa cells (Supplementary Figure S2; white bars), were intoxicated with mPA83-EGF plus LF_N_-DTA. Importantly, a decrease in cell viability in both cell lines was obvious with 20% (v/v) DMSO or when cells were intoxicated with positive controls (10 nM native diphtheria toxin (DT) and the combinations of 20 nM PA83 or 20 nM PA63 plus 10 nM LF_N_-DTA, respectively), but not with negative controls (the combination of 20 nM mPA83 alone plus 10 nM LF_N_-DTA or the single toxin components) (Supplementary Figure S2).

These results demonstrate that cell specificity of the CRISPA approach can be achieved by the use of a mutated PA variant fused to a cell surface-specific ligand.

## Discussion

CRISPR/Cas9 genome engineering requires the formation of a gRNA:Cas9 complex directly at the target gene in the cell nucleus. Nowadays, a plethora of methods are available for the viral or non-viral delivery of both components into target cells, each with its own specific advantages and disadvantages (Liu et al., 2017). Here, we describe a novel, modular CRISPA approach for the transient, non-viral delivery of Cas9 protein into mammalian cells via the transport machinery (PA) of the anthrax toxin.

As proof-of-concept for this CRISPA approach, we knocked out the gene encoding LSR in HCT116 colon cancer cells. Besides its physiological roles in lipoprotein uptake and stability of tricellular junctions, LSR acts also as entry receptor for the *C. difficile* binary toxin CDT (Papatheodorou et al., 2011, Papatheodorou et al., 2018). The latter helped us in selecting homozygous/biallelic CRISPA-derived, LSR-deficient HCT116 cells that are resistant to CDT. CRISPA introduced a nucleotide insertion (frameshift mutation) exactly at the Cas9 cleavage site, targeted by the LSR-specific gRNA. Transfer of the CRISPA approach to 293T cells stably expressing GFP, indicates that the CRISPA approach may be applicable to a wide range of target cells and genes. In order to reach cell specificity, the receptor-binding domain of PA has to be replaced with the respective protein ligand of a given cell surface-marker.

We used two different recombinant Cas9 variants for PA-mediated entry into target cells. One of them already carried a His6-tag at its N-terminus and the other Cas9 variant (with a His6-tag at its C-terminus) was fused to the N-terminus of the anthrax lethal factor (LF_N_). Notably, the His6-tag is very convenient for purification of recombinant Cas9 proteins and, if preferred, various His6-tagged Cas9 variants are commercially available. Both N-terminal sequences (His6-tag and LF_N_) enable the interaction with and translocation of cargo proteins through the PA pore formed in endosomal membranes upon acidification of the endosomal lumen (Rabideau and Pentelute, 2016). In our hands, both N-termini showed similar efficiencies in knocking out GFP in 293T-GFP cells, as estimated by a T7 endonuclease I mismatch detection assay.

The use of mPA83-EGF instead of PA in our CRISPA approach would thus enable a selective genome engineering in EGFR-expressing cells. Accordingly, fusions of mPA83 with other receptor ligands will allow CRISPA to adapt to specific cell types. Other non-viral and transient CRISPR/Cas9 delivery systems, such as cell penetrating peptides, lack this possibility. Verdurmen and others succeeded in retargeting mPA83 to epithelial adhesion molecule (EpCAM)-expressing cells by fusion with an EpCAM-targeting designed ankyrin repeat protein (DARPin) (Verdurmen et al., 2015). Such cell-specific CRISPA approaches would pave the way for *in vivo* clinical applications.

It has been shown by several groups that *Streptococcus pyogenes* Cas9 does not dissociate from the cleaved DNA due to an extremely slow rate of turnover (Sternberg et al., 2014; Richardson et al., 2016; Jones et al., 2017; Gong et al., 2018; Raper et al., 2018). We therefore speculate that this prominent feature of *S. pyogenes* Cas9 might explain the rather low gene-editing efficiency that we calculated via the T7EI assay for our CRISPA approach in dividing cells. Yourik and others reported the first multiple-turnover Cas9 from *Staphylococcus aureus* and speculated that a faster rate of turnover by *S. aureus* Cas9 should result in more attempts to re-introduce the double-stranded DNA break at the site of interest and thus might result in a higher degree of editing (Yourik et al., 2019). We are planning to test this Cas9 ortholog in a follow-up study, whether it is more efficient in gene-editing after delivery into cells via PA.

Inefficient unfolding prior and/or refolding after transport of Cas9 into the cytosol, as well as quick degradation of Cas9 in the cell might also contribute to the rather low gene-editing efficiency of our CRISPA approach. However, the CRISPA approach should also function with hyper-accurate Cas9 variants or even with Cas9 alternatives, such as Cpf1, Cas12a and Cas12b, and smaller Cas9 orthologues (e.g. CjCas9) (Broeders et al., 2020). Future studies are needed to clarify, whether smaller or stabilized Cas9 variants might increase the efficiency of the CRISPA approach. In addition, these Cas9 variations might help to reduce off-target effects.

Transient and non-viral delivery of gRNA molecules into cells remains a challenge. One option is the *in vitro* assembly of ribonuclearprotein (RNP) complexes between the Cas9 protein and the guide RNA (DeWitt et al., 2017). However, such Cas9-gRNA-RNPs are delivered transiently into cells for instance via electroporation, which is rather unspecific and not applicable in a clinical setting. However, it was reported that Cas9 protein and single guide RNA can be coupled to cell-penetrating peptides (CPPs) to yield RNP nanoparticles, which led to efficient gene disruption in treated cells (Ramakrishna et al., 2014). In comparison, the CRISPA approach most likely lacks the possibility to transport such RNP nanoparticles into cells, because the translocation across the channel formed by PA in endosomes requires unfolding of cargo proteins.

However, a disarmed anthrax toxin was used by Dyer and others for the cytosolic delivery of antisense oligonucleotides (ASOs) and siRNA (Dyer et al., 2015). Transport of ASOs or siRNA via PA was achieved via LF_N_ fusions of either *Saccharomyces cerevisiae* GAL4 or human protein kinase R (PKR), both of which are capable of binding dsDNA or dsRNA, respectively. But it remained unclear, how the ASOs and siRNA were transported through the channel, given the fact that the pore diameter of the channel is too small to allow the transport of GAL4:dsDNA or PKR:dsRNA complexes in an unfolded state.

Nevertheless, piggyback-transport of sgRNA through the PA channel via a sgRNA-binding protein fused to LF_N_ would allow simultaneous delivery of Cas9 and sgRNA into target cells. However, in its current state, the CRISPA approach needs to be combined with other viral or non-viral methods that are capable of delivering the single guide RNA into cells. Notably, a transient toxin-based siRNA delivery strategy was developed just recently on the basis of an attenuated diphtheria toxin (Arnold et al., 2020).

The approach presented in this study represents an additional step towards safe translation of the powerful CRISPR/Cas9 genome engineering technology into clinics, especially with respect to *ex vivo* applications. Using CRISPA cell-type specificity in transient systems may reduce off-target effects. Moreover, the CRISPA approach provides another example of the power of bacterial protein toxins as cellular transport systems and potential therapeutic agents.

## Data Availability

The raw data supporting the conclusions of this article will be made available by the authors, without undue reservation.

## References

[B1] Arnold A. E. Smith L. J. Beilhartz G. L. Bahlmann L. C. Jameson E. Melnyk R. A. (2020). Attenuated Diphtheria Toxin Mediates siRNA Delivery. Sci. Adv. 6. 10.1126/sciadv.aaz4848 PMC7195190 32917630

[B2] Arora N. Leppla S. H. (1994). Fusions of Anthrax Toxin Lethal Factor with Shiga Toxin and Diphtheria Toxin Enzymatic Domains Are Toxic to Mammalian Cells. Infect. Immun. 62, 4955–4961. 10.1128/IAI.62.11.4955-4961.1994 7927776 PMC303212

[B3] Arora N. Leppla S. H. (1993). Residues 1-254 of Anthrax Toxin Lethal Factor Are Sufficient to Cause Cellular Uptake of Fused Polypeptides. J. Biol. Chem. 268, 3334–3341. 10.1016/s0021-9258(18)53698-x 8429009

[B4] Barth H. Blocker D. Behlke J. Bergsma-Schutter W. Brisson A. Benz R. (2000). Cellular Uptake of Clostridium Botulinum C2 Toxin Requires Oligomerization and Acidification. J. Biol. Chem. 275, 18704–18711. 10.1074/jbc.M000596200 10749859

[B5] Beilhartz G. L. Sugiman-Marangos S. N. Melnyk R. A. (2017). Repurposing Bacterial Toxins for Intracellular Delivery of Therapeutic Proteins. Biochem. Pharmacol. 142, 13–20. 10.1016/j.bcp.2017.04.009 28408344

[B6] Beitzinger C. Stefani C. Kronhardt A. Rolando M. Flatau G. Lemichez E. (2012). Role of N-Terminal His6-Tags in Binding and Efficient Translocation of Polypeptides into Cells Using Anthrax Protective Antigen (PA). PLoS One 7, e46964. 10.1371/journal.pone.0046964 23056543 PMC3466187

[B7] Blanke S. R. Milne J. C. Benson E. L. Collier R. J. (1996). Fused Polycationic Peptide Mediates Delivery of Diphtheria Toxin A Chain to the Cytosol in the Presence of Anthrax Protective Antigen. Proc. Natl. Acad. Sci. U. S. A. 93, 8437–8442. 10.1073/pnas.93.16.8437 8710889 PMC38689

[B8] Blöcker D. Behlke J. Aktories K. Barth H. (2001). Cellular Uptake of the *Clostridium perfringens* Binary iota-toxin. Infect. Immun. 69, 2980–2987. 10.1128/IAI.69.5.2980-2987.2001 11292715 PMC98251

[B9] Broeders M. Herrero-Hernandez P. Ernst M. P. T. van der Ploeg A. T. Pijnappel W. W. M. P. (2020). Sharpening the Molecular Scissors: Advances in Gene-Editing Technology. iScience 23, 100789. 10.1016/j.isci.2019.100789 31901636 PMC6941877

[B10] Chavarría-Smith J. Vance R. E. (2013). Direct Proteolytic Cleavage of NLRP1B Is Necessary and Sufficient for Inflammasome Activation by Anthrax Lethal Factor. Plos Pathog. 9, e1003452. 10.1371/journal.ppat.1003452 23818853 PMC3688554

[B11] Corish P. Tyler-Smith C. (1999). Attenuation of green Fluorescent Protein Half-Life in Mammalian Cells. Protein Eng. 12, 1035–1040. 10.1093/protein/12.12.1035 10611396

[B12] DeWitt M. A. Corn J. E. Carroll D. (2017). Genome Editing via Delivery of Cas9 Ribonucleoprotein. Methods 121-122, 9–15. 10.1016/j.ymeth.2017.04.003 28410976 PMC6698184

[B13] Dull T. Zufferey R. Kelly M. Mandel R. J. Nguyen M. Trono D. (1998). A Third-Generation Lentivirus Vector with a Conditional Packaging System. J. Virol. 72, 8463–8471. 10.1128/jvi.72.11.8463-8471.1998 9765382 PMC110254

[B14] Dyer P. D. R. Shepherd T. R. Gollings A. S. Shorter S. A. Gorringe-Pattrick M. A. M. Tang C. K. (2015). Disarmed Anthrax Toxin Delivers Antisense Oligonucleotides and siRNA with High Efficiency and Low Toxicity. J. Control Release 220, 316–328. 10.1016/j.jconrel.2015.10.054 26546271

[B15] Ernst K. Schmid J. Beck M. Hägele M. Hohwieler M. Hauff P. (2017). Hsp70 Facilitates Trans-membrane Transport of Bacterial ADP-Ribosylating Toxins into the Cytosol of Mammalian Cells. Sci. Rep. 7, 2724. 10.1038/s41598-017-02882-y 28578412 PMC5457432

[B16] Gong S. Yu H. H. Johnson K. A. Taylor D. W. (2018). DNA Unwinding Is the Primary Determinant of CRISPR-Cas9 Activity. Cell Rep 22, 359–371. 10.1016/j.celrep.2017.12.041 29320733 PMC11151164

[B17] Hemmasi S. Czulkies B. A. Schorch B. Veit A. Aktories K. Papatheodorou P. (2015). Interaction of the *Clostridium difficile* Binary Toxin CDT and its Host Cell Receptor, Lipolysis-Stimulated Lipoprotein Receptor (LSR). J. Biol. Chem. 290, 14031–14044. 10.1074/jbc.M115.650523 25882847 PMC4447975

[B18] Hirakawa M. P. Krishnakumar R. Timlin J. A. Carney J. P. Butler K. S. (2020). Gene Editing and CRISPR in the Clinic: Current and Future Perspectives. Biosci. Rep. 40. 10.1042/BSR20200127 PMC7146048 32207531

[B19] Jones D. L. Leroy P. Unoson C. Fange D. Ćurić V. Lawson M. J. (2017). Kinetics of dCas9 Target Search in *Escherichia coli* . Science 357, 1420–1424. 10.1126/science.aah7084 28963258 PMC6150439

[B20] Kaiser E. Kroll C. Ernst K. Schwan C. Popoff M. Fischer G. (2011). Membrane Translocation of Binary Actin-ADP-Ribosylating Toxins from *Clostridium difficile* and *Clostridium perfringens* Is Facilitated by Cyclophilin A and Hsp90. Infect. Immun. 79, 3913–3921. 10.1128/IAI.05372-11 21768281 PMC3187244

[B21] Koepke L. Winter B. Grenzner A. Regensburger K. Engelhart S. van der Merwe J. A. (2020). An Improved Method for High-Throughput Quantification of Autophagy in Mammalian Cells. Sci. Rep. 10, 12241. 10.1038/s41598-020-68607-w 32699244 PMC7376206

[B22] Lang A. E. Schmidt G. Schlosser A. Hey T. D. Larrinua I. M. Sheets J. J. (2010). Photorhabdus Luminescens Toxins ADP-Ribosylate Actin and RhoA to Force Actin Clustering. Science 327, 1139–1142. 10.1126/science.1184557 20185726

[B23] Lino C. A. Harper J. C. Carney J. P. Timlin J. A. (2018). Delivering CRISPR: a Review of the Challenges and Approaches. Drug Deliv. 25, 1234–1257. 10.1080/10717544.2018.1474964 29801422 PMC6058482

[B24] Liu C. Zhang L. Liu H. Cheng K. (2017). Delivery Strategies of the CRISPR-Cas9 Gene-Editing System for Therapeutic Applications. J. Control Release 266, 17–26. 10.1016/j.jconrel.2017.09.012 28911805 PMC5723556

[B25] Mali P. Yang L. Esvelt K. M. Aach J. Guell M. DiCarlo J. E. (2013). RNA-guided Human Genome Engineering via Cas9. Science 339, 823–826. 10.1126/science.1232033 23287722 PMC3712628

[B26] Marr R. A. Guan H. Rockenstein E. Kindy M. Gage F. H. Verma I. (2004). Neprilysin Regulates Amyloid Beta Peptide Levels. J. Mol. Neurosci. 22, 5–11. 10.1385/JMN:22:1-2:5 14742905

[B27] Mechaly A. McCluskey A. J. Collier R. J. (2012). Changing the Receptor Specificity of Anthrax Toxin. MBio 3. 10.1128/mBio.00088-12 PMC3569862 22550037

[B28] Milne J. C. Blanke S. R. Hanna P. C. Collier R. J. (1995). Protective Antigen-Binding Domain of Anthrax Lethal Factor Mediates Translocation of a Heterologous Protein Fused to its Amino- or Carboxy-Terminus. Mol. Microbiol. 15, 661–666. 10.1111/j.1365-2958.1995.tb02375.x 7783638

[B29] Moayeri M. Leppla S. H. (2009). Cellular and Systemic Effects of Anthrax Lethal Toxin and Edema Toxin. Mol. Aspects Med. 30, 439–455. 10.1016/j.mam.2009.07.003 19638283 PMC2784088

[B30] Moayeri M. Leppla S. H. Vrentas C. Pomerantsev A. P. Liu S. (2015). Anthrax Pathogenesis. Annu. Rev. Microbiol. 69, 185–208. 10.1146/annurev-micro-091014-104523 26195305

[B31] Nelson C. E. Gersbach C. A. (2016). Engineering Delivery Vehicles for Genome Editing. Annu. Rev. Chem. Biomol. Eng. 7, 637–662. 10.1146/annurev-chembioeng-080615-034711 27146557

[B32] Papatheodorou P. Barth H. Minton N. Aktories K. (2018). Cellular Uptake and Mode-Of-Action of *Clostridium difficile* Toxins. Adv. Exp. Med. Biol. 1050, 77–96. 10.1007/978-3-319-72799-8_6 29383665

[B33] Papatheodorou P. Carette J. E. Bell G. W. Schwan C. Guttenberg G. Brummelkamp T. R. (2011). Lipolysis-stimulated Lipoprotein Receptor (LSR) Is the Host Receptor for the Binary Toxin *Clostridium difficile* Transferase (CDT). Proc. Natl. Acad. Sci. U. S. A. 108, 16422–16427. 10.1073/pnas.1109772108 21930894 PMC3182710

[B34] Papatheodorou P. Hornuss D. Nölke T. Hemmasi S. Castonguay J. Picchianti M. (2013). *Clostridium difficile* Binary Toxin CDT Induces Clustering of the Lipolysis-Stimulated Lipoprotein Receptor into Lipid Rafts. MBio 4, e00244–13. 10.1128/mBio.00244-13 23631918 PMC3648903

[B35] Piot N. van der Goot F. G. Sergeeva O. A. (2021). Harnessing the Membrane Translocation Properties of AB Toxins for Therapeutic Applications. Toxins (Basel) 13, 36. 10.3390/toxins13010036 33418946 PMC7825107

[B36] Rabideau A. E. Pentelute B. L. (2016). Delivery of Non-native Cargo into Mammalian Cells Using Anthrax Lethal Toxin. ACS Chem. Biol. 11, 1490–1501. 10.1021/acschembio.6b00169 27055654

[B37] Ramakrishna S. Kwaku Dad A. B. Beloor J. Gopalappa R. Lee S. K. Kim H. (2014). Gene Disruption by Cell-Penetrating Peptide-Mediated Delivery of Cas9 Protein and Guide RNA. Genome Res. 24, 1020–1027. 10.1101/gr.171264.113 24696462 PMC4032848

[B38] Raper A. T. Stephenson A. A. Suo Z. (2018). Functional Insights Revealed by the Kinetic Mechanism of CRISPR/Cas9. J. Am. Chem. Soc. 140, 2971–2984. 10.1021/jacs.7b13047 29442507

[B39] Richardson C. D. Ray G. J. DeWitt M. A. Curie G. L. Corn J. E. (2016). Enhancing Homology-Directed Genome Editing by Catalytically Active and Inactive CRISPR-Cas9 Using Asymmetric Donor DNA. Nat. Biotechnol. 34, 339–344. 10.1038/nbt.3481 26789497

[B40] Schneider C. A. Rasband W. S. Eliceiri K. W. (2012). NIH Image to ImageJ: 25 Years of Image Analysis. Nat. Methods 9, 671–675. 10.1038/nmeth.2089 22930834 PMC5554542

[B41] Schwan C. Nölke T. Kruppke A. S. Schubert D. M. Lang A. E. Aktories K. (2011). Cholesterol- and Sphingolipid-Rich Microdomains Are Essential for Microtubule-Based Membrane Protrusions Induced by *Clostridium difficile* Transferase (CDT). J. Biol. Chem. 286, 29356–29365. 10.1074/jbc.M111.261925 21705797 PMC3190741

[B42] Sharma O. Collier R. J. (2014). Polylysine-mediated Translocation of the Diphtheria Toxin Catalytic Domain through the Anthrax Protective Antigen Pore. Biochemistry 53, 6934–6940. 10.1021/bi500985v 25317832 PMC4230326

[B43] Singh Y. Chaudhary V. K. Leppla S. H. (1989). A Deleted Variant of Bacillus Anthracis Protective Antigen Is Non-toxic and Blocks Anthrax Toxin Action *In Vivo* . J. Biol. Chem. 264, 19103–19107. 10.1016/s0021-9258(19)47273-6 2509473

[B44] Sternberg S. H. Redding S. Jinek M. Greene E. C. Doudna J. A. (2014). DNA Interrogation by the CRISPR RNA-Guided Endonuclease Cas9. Nature 507, 62–67. 10.1038/nature13011 24476820 PMC4106473

[B45] Sterthoff C. Lang A. E. Schwan C. Tauch A. Aktories K. (2010). Functional Characterization of an Extended Binding Component of the Actin-ADP-Ribosylating C2 Toxin Detected in Clostridium Botulinum Strain (C) 2300. Infect. Immun. 78, 1468–1474. 10.1128/IAI.01351-09 20145093 PMC2849413

[B46] Tonello F. Naletto L. Romanello V. Dal Molin F. Montecucco C. (2004). Tyrosine-728 and Glutamic Acid-735 Are Essential for the Metalloproteolytic Activity of the Lethal Factor of Bacillus Anthracis. Biochem. Biophys. Res. Commun. 313, 496–502. 10.1016/j.bbrc.2003.11.134 14697216

[B47] van der Goot G. Young J. A. (2009). Receptors of Anthrax Toxin and Cell Entry. Mol. Aspects Med. 30, 406–412. 10.1016/j.mam.2009.08.007 19732789 PMC2783407

[B48] Verdurmen W. P. Luginbühl M. Honegger A. Plückthun A. (2015). Efficient Cell-specific Uptake of Binding Proteins into the Cytoplasm through Engineered Modular Transport Systems. J. Control Release 200, 13–22. 10.1016/j.jconrel.2014.12.019 25526701

[B49] Wang L. Zheng W. Liu S. Li B. Jiang X. (2019). Delivery of CRISPR/Cas9 by Novel Strategies for Gene Therapy. Chembiochem 20, 634–643. 10.1002/cbic.201800629 30393919

[B50] Young J. A. Collier R. J. (2007). Anthrax Toxin: Receptor Binding, Internalization, Pore Formation, and Translocation. Annu. Rev. Biochem. 76, 243–265. 10.1146/annurev.biochem.75.103004.142728 17335404

[B51] Yourik P. Fuchs R. T. Mabuchi M. Curcuru J. L. Robb G. B. (2019). *Staphylococcus aureus* Cas9 Is a Multiple-Turnover Enzyme. Rna 25, 35–44. 10.1261/rna.067355.118 30348755 PMC6298560

[B52] Zahaf N. I. Lang A. E. Kaiser L. Fichter C. D. Lassmann S. McCluskey A. (2017). Targeted Delivery of an ADP-Ribosylating Bacterial Toxin into Cancer Cells. Sci. Rep. 7, 41252. 10.1038/srep41252 28128281 PMC5269596

[B53] Zhang F. Wang S. Yin L. Yang Y. Guan Y. Wang W. (2015). Quantification of Epidermal Growth Factor Receptor Expression Level and Binding Kinetics on Cell Surfaces by Surface Plasmon Resonance Imaging. Anal. Chem. 87, 9960–9965. 10.1021/acs.analchem.5b02572 26368334 PMC4836855

[B54] Zuris J. A. Thompson D. B. Shu Y. Guilinger J. P. Bessen J. L. Hu J. H. (2015). Cationic Lipid-Mediated Delivery of Proteins Enables Efficient Protein-Based Genome Editing *In Vitro* and *In Vivo* . Nat. Biotechnol. 33, 73–80. 10.1038/nbt.3081 25357182 PMC4289409

