# Supplementary material for: CRISPA: A Non-viral, Transient Cas9 Delivery System Based on Reengineered Anthrax Toxin
Source: Front Pharmacol. 2021 Oct 18;12:770283. doi: 10.3389/fphar.2021.770283 (PMC8558532; doi:10.3389/fphar.2021.770283)
Supplement: Supplementary file 1 [file DataSheet1.pdf]

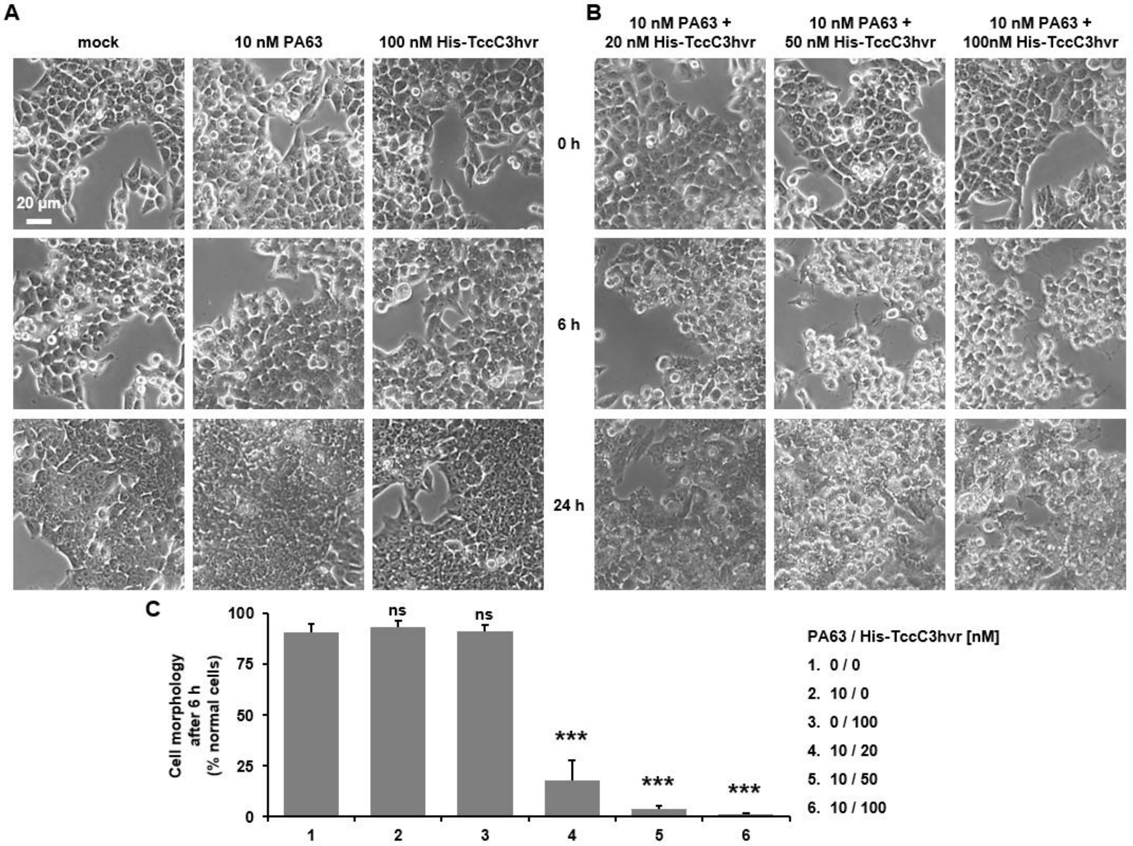

Figure S1. Intoxication of HCT116 cells with PA63 and His-TccC3hvr. (A, B) HCT116 cells were incubated (A) without any protein (mock), with 10 nM PA63 or with 100 nM His-TccC3hvr or (B) with a combination of 10 nM PA63 plus 20, 50 and 100 nM His-TccC3-hvr, respectively. The intoxication process was monitored microscopically after 0, 6 and 24 h. (C) Bar diagram is the quantification of the images shown in (A, B) and shows the total percentage of cells with normal morphology after 6 h of intoxication (n=3, ±SD, ***p<0.001, ns = not significant). (A, B) A scale bar representative for all images is shown in the upper left image of (A).

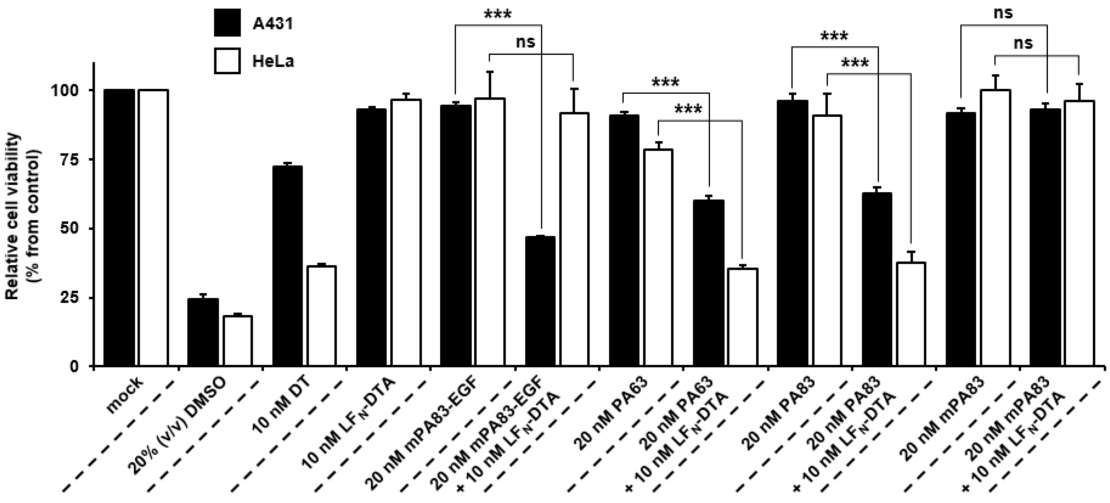

Figure S2. Cell-specific intoxication of A431 and HeLa cells with mPA83-EGF. Bar diagram shows the relative cell viability of A431 (black bars) and HeLa cells (white bars) after 24 h of incubation with the indicated toxin combinations or the single toxin components. Treatment of cells with 20% (v/v) DMSO served as positive control for the MTS-based cell viability assay. Results shown are mean values calculated from three samples performed in parallel and with error bars indicating n=3 ±SD (***p<0.001; ns, not significant). Values are normalized to the cell viability mean values of mock-treated A431 and HeLa cells, respectively, which were set to 100%. Experiment was performed at least twice with identical results.
